# Examining the Effect of the Affordable Care Act on Two Illinois Emergency Departments

**DOI:** 10.5811/westjem.2019.6.41943

**Published:** 2019-08-06

**Authors:** Beatrice D. Probst, Luther Walls, Michael Cirone, Talar Markossian

**Affiliations:** * Loyola University Chicago, Stritch School of Medicine, Department of Emergency Medicine, Maywood, Illinois; † University of Illinois at Chicago, Advocate Christ Medical Center, Department of Emergency Medicine, Oak Lawn, Illinois; ‡ Public Health Sciences at Loyola University Chicago, Maywood, Illinois

## Abstract

**Introduction:**

The emergency department (ED) has long served as a safety net for the uninsured and those with limited access to routine healthcare. This study aimed to compare the characteristics and severity of ED visits in an Illinois academic medical center (AMC) and community hospital (CH) of a single health system before and after the implementation of the Affordable Care Act (ACA).

**Methods:**

This was a retrospective record review of 357,764 ED visits from January 1, 2011–December 31, 2016, of which 74% were at the AMC and 26% at the CH. We assessed the severity of ED visits by applying the previously validated Ballard algorithm, which classifies ED visits as non-emergent, intermediate, or emergent. Descriptive analyses were conducted to compare the characteristics of ED visits before and after the implementation of the ACA. We conducted multilevel logistic regression analysis to examine the odds of non-emergent compared to intermediate/emergent ED visits by the ACA implementation status controlling for patient demographic characteristics, insurance status, and multiple visits per patient.

**Results:**

ED visits for patients with Medicaid or other governmental coverages increased in the post-ACA compared to pre-ACA period (Pre: 33.2 % vs Post: 38.3% at the AMC, and Pre: 29.7% vs Post: 35.1% at the CH). A statistically significant decrease in ED visits for uninsured patients was observed at the AMC and CH in the post-ACA period compared to the pre-ACA period (Pre: 12.1% vs Post: 6.4%, and Pre: 13.9% vs Post: 9.8%, respectively). Results from the regression analysis showed a significant decreased odds of non-emergent vs intermediate/emergent ED visits during the post-ACA period compared to the pre-ACA period at the AMC (odds ratio [OR] 0.68; confidence interval [CI], 0.66–0.70). However, an increased odds of non-emergent vs. intermediate/emergent ED visits was observed at the CH (OR 1.09; CI, 1.04–1.14).

**Conclusion:**

Similar to other Medicaid expansion states, ED utilization for uninsured patients decreased at both the AMC and the CH in the post-ACA period. While Medicaid visits for children < 18 years declined in the post-ACA period, it increased for ages 21 to 65 years of age. Contrary to our hypothesis, the severity of emergent ED visits increased in the post-ACA period but not at the CH.

## INTRODUCTION

The emergency department (ED) has long served as a safety net for the uninsured and those with limited access to routine healthcare. In recent years, ED crowding has worsened as patients who lack timely access to primary care have used the ED for non-emergent conditions. Inappropriate ED utilization can result in unnecessary testing, procedures, and admissions, all of which may contribute to rising healthcare costs. The Affordable Care Act (ACA) of 2010 aimed to improve access to primary care providers for non-emergent complaints by providing expanded insurance coverage options. In 2013, Illinois opted to expand Medicaid to low-income adults resulting in a net increase in Medicaid coverage of more than 486,000 individuals in the first three years after implementation of the ACA.^1^,^2^ Despite these efforts, studies measuring ED utilization before and after the enactment of the ACA have yielded mixed results.^3^–^5^ Estimates of the effect of health insurance coverage on ED visits is a complex relationship that must be factored with out-of-pocket expenses to patients and access to alternative sources of healthcare, as well as reimbursement to primary care providers. Economic theory suggests that expanding access to health insurance could either increase or reduce ED use.^6^

Prior to the implementation of the ACA, an independently validated ED algorithm that classifies ED visits according to the severity of the visit was created to analyze and predict ED utilization patterns.^7^,^8^ Applying the Ballard algorithm to analyze patterns in ED utilization before and after the ACA implementation could enhance the growing body of literature about understanding the impact of the ACA implementation. Results may guide future health policy legislation regarding strategies for alternative healthcare utilization, payor options, market place directions, and resource allocation.

This retrospective study compared the characteristics and severity of a single, suburban Illinois health system’s ED visits. We assessed the severity of ED visits by applying the Ballard algorithm, which classifies ED visits to non-emergent, intermediate, and emergent. We hypothesized that similar to other Medicaid expansion states, the EDs would see an increase in the percentage of ED patients who were insured after ACA implementation but that the severity of ED visits would not be impacted as emergent conditions were likely still to require ED care. The primary outcome variable in this study was the severity of ED visits relative to implementation of the ACA. Secondary outcome variables included the characteristics of patients and ED visits.

## METHODS

We performed a retrospective record review of ED visits from a single health system’s electronic health record (EHR). The study comprised a Level 1 academic medical center (AMC) in Maywood, IL, and a Level 2 community hospital (CH) four miles away in Melrose Park, IL, before and after the implementation of the ACA. Neither ED has an affiliated emergency medicine residency, but the AMC supports residents from other core specialties. We electronically extracted the data from the clinical data warehouse, where data from the EHR resides and is refreshed nightly. The variables definition sheet was prepared by the investigators and provided to the health system’s senior programmer, who performed the data extractions. The extracted data was reviewed by the study investigators for any inconsistencies and validated by chart reviews by the emergency physician on the team in a random sample of ED visits (~50 patients) to validate that the data pulled electronically met the variables definitions. The timeline of the AMC data query was from January 1, 2011–December 31, 2016, and the CH from January 1, 2013–December 31, 2016. The CH’s query was limited to the period when electronic data from the EHR was available. A pre-ACA period was defined from January 1, 2011–December 31, 2013, and a post-ACA period from April 1, 2014–December 31, 2016. We excluded ED visits from January 1, 2014–March 31, 2014, from this study to avoid uncertainties around the ACA open enrollment period. We also excluded from the analysis all visits in which patients left without being seen. The study was reviewed and approved by the health system’s institutional review board.

**Population Health Research Capsule**

What do we already know about this issue?

*After Affordable Care Act implementation in Medicaid expansion states such as Illinois, emergency departments (EDs) experienced an increase in visits, primarily for insured patients*.

What was the research question?

*This study examined the severity of ED visits of a single health system by application of the Billings-Ballard algorithm*.

What was the major finding of the study?

*Visit increases at the academic medical center were classified as emergent compared with non-emergent at the community hospital*.

How does this improve population health?

*Variances in ED use across a single health system highlight the need to develop strategies for non-emergent patient access and alternative resources for emergent patients*.

We used the Ballard algorithm to classify the ED visits into emergent, non-emergent, or intermediate based on the discharging *International Classification of Diseases*, 9^th^ and 10^th^ revisions (ICD-9/10) diagnosis codes. The unclassified category in this study included uncommon diagnoses and diagnoses of mental health, injuries, and substance and alcohol abuse. The focus of the Billings and later revised Ballard algorithms were to identify ED visits that could have been preventable by appropriate primary care. The original Billings algorithm assigned the probability that each ED visit ICD-9/10 diagnosis code fell into one of four severity categories: non-emergent (NE), primary care treatable emergency (PCT), a preventable or avoidable emergency not treatable in an office visit (EPA), and an emergency that is not preventable or avoidable (ENPA). The algorithm excludes uncommon diagnoses and treats mental health, injuries, and substance and alcohol abuse diagnosis separately.

In the revised Ballard algorithm, the probabilities derived from the ICD-9/10 diagnosis code were used to classify each visit as non-emergent or emergent using the dominant probability, or intermediate when there was 50% probability of being both emergent and non-emergent. NE and PCT were considered non-emergent, and EPA and ENPA were considered emergent. Each ED visit was then classified as emergent or non-emergent using the classification of the most emergent diagnosis. For example, in the Ballard algorithm, infectious colitis has 100% probability of being non-emergent. Cardiac dysrhythmia has 13% probability of being non-emergent and 88% probability of being emergent; therefore, it is classified as emergent. Hypertensive chronic kidney disease has 79% probability of being non-emergent and 21% probability of being emergent; therefore, it is classified as non-emergent. 7,8

We conducted descriptive univariate analyses for proportions and bivariate comparisons using the chi-squared test for categorical variables and conducted the t-test for continuous variables to compare the characteristics of ED visits before and after ACA implementation. The severity of visits was compared before and after implementation of the ACA by location of visits. We conducted multilevel logistic regression analysis to examine the odds of non-emergent ED visit compared to intermediate/emergent ED visits by the ACA implementation status, with unclassified ED visits by the Ballard algorithm excluded from the regression analyses. The analyses controlled for patient demographic characteristics and insurance status. Multiple visits per patient were adjusted in the regression analysis with a random effect term. All statistical tests were two-sided and a P<0.05 was considered statistically significant. We conducted all analyses using the Stata 15.1 (College Station, TX) statistical software.

## RESULTS

There were 357,764 ED visits during the study period, of which 74% were at the AMC and 26% at the CH. Patients’ demographic characteristics and insurance status differed significantly between pre- and post-ACA periods at the AMC and CH. When compared to the pre-ACA period, AMC and CH ED visits for children < 18 years decreased in the post-ACA period (Pre: 24.7% vs Post: 22.3%, and Pre: 17.5% vs. Post: 16.0%, respectively), while AMC and the CH ED visits for ages 40 to 64.9 years increased post-ACA (Pre: 28.9% vs Post: 30.9%, and Pre: 28.7% vs Post: 29.5%, respectively). AMC ED visits for Black patients decreased post-ACA (Pre: 39.8% vs Post: 36.2%), while visits for Hispanic patients increased (Pre: 21.7% vs Post: 24.8%). AMC and CH ED visits for patients with Medicaid or other governmental coverages increased in the post-ACA (Pre: 33.2% vs Post: 38.3%, and Pre: 29.7% vs Post: 35.1%, respectively). Uninsured patients accounted for a statistically significant decrease in AMC and CH ED visits in the post-ACA period (Pre: 12.1% vs Post: 6.4%, and Pre: 13.9% vs. Post: 9.8%, respectively).

Also compared to pre-ACA, at the AMC the proportion of Medicaid ED visits for children younger than 18 years decreased significantly post-ACA (Pre: 51.3% vs Post: 39.1%). Conversely, the percentage of AMC ED Medicaid visits in the older age brackets all increased in the post-ACA period, from Pre: 7.5% vs Post: 8.8% (21 to 25.9 years), Pre: 18.1% vs Post: 21.0% (26 to < 39.9 years), and Pre: 16.8% vs Post: 24.5% (40 < 65 years).

The mean number of ED visits per patient declined at the AMC (Pre: 1.41 vs Post: 1.35) and at the CH (Pre: 1.38 vs. Post: 1.36) from the pre- compared to post-ACA period (Table 1). At the AMC, compared to the pre-ACA period there was a statistically significant increase in the ED visits that resulted in hospitalization during post-ACA (Pre: 32.1 % vs Post: 35.0%). At the CH, compared with the pre-ACA period, the post-ACA period saw a statistically significant decline in the ED visits that resulted in hospitalization (Pre: 29.4% vs Post: 28.2%) (Table 2). Readmissions within 48 hours and one week were not statistically different from pre- to post-ACA period at the AMC and CH.

**Figure 1:**
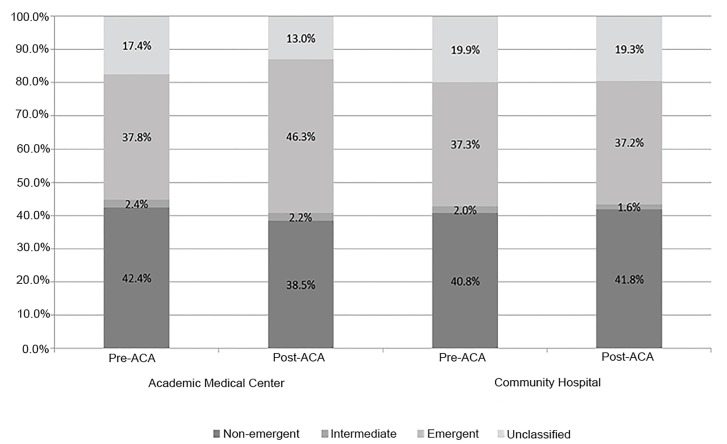
The severity of emergency department visits according to the Ballard algorithm.

During the study period, the distribution of the severity of AMC ED visits for emergent and non-emergent visits varied significantly (Figure 1). Compared to pre-ACA, a higher percent of ED visits at the AMC were emergent post-ACA (Pre: 37.8% vs Post: 46.3%), while conversely there was a decline in the non-emergent visits (Pre: 42.4% vs Post: 38.5%). The CH did not experience similar changes in the categories of emergent and non-emergent visits.

Results from the regression analysis showed significantly decreased odds of non-emergent vs intermediate/emergent ED visits during the post-ACA period compared to the pre-ACA period at the AMC across all payor groups (odds ratio (OR) 0.68, confidence interval (CI), 0.66–0.70). However, an increased odds of non-emergent vs intermediate/emergent ED visits was observed at the CH (OR 1.09; CI, 1.04–1.14) (Table 3). Results were similar when the analysis was repeated for the odds of non-emergent vs emergent only, excluding intermediate ED visits from the analysis. Stratified regression analysis by insurance status showed similar results; however, notably, the odds of non-emergent visits increased significantly during post-ACA in Medicare and uninsured patients in the CH.

## DISCUSSION

Recent studies have yielded mixed conclusions in evaluating changes in ED utilization following ACA implementation in Medicaid expansion states.^9^–^13^ Several studies have shown no significant increase in ED visits, while others (including studies in Illinois) demonstrated increased ED utilization following ACA implementation.^12^,^13^ While there is a growing body of literature comparing ED volumes and payor mixes in the pre- and post-ACA periods, to our knowledge none have investigated changes in the severity of ED visits. In Illinois, over 600,000 people have enrolled in Medicaid since expansion in 2014, with Medicaid and the Children’s Health Insurance Program now covering 2.9 of the 12.8 million citizens.^1^ However, there is only one primary care physician (PCP) for every 1462 citizens, one of the lowest ratios nationally.^14^ Although many patients gained insurance coverage as a result of the ACA, access to healthcare remains an obstacle. Many theorized that increasing insurance coverage without a significant increase in PCPs could overwhelm EDs with non-emergent visits in patients empowered by their new insurance status to seek medical care but unable to obtain timely, primary care appointments.

Given the inconsistencies in the literature, we sought to examine the severity of ED visits pre- and post-ACA implementation periods in an AMC and a CH located in a major urban area within a Medicaid expansion state. Similar to other studies, our results demonstrated that utilization for uninsured patients decreased at both the AMC and the CH in the post-ACA period. While Medicaid visits for children < 18 years declined in the post-ACA period, it increased for ages 21 to 65 years of age. Children’s healthcare needs often involve wellness and routine immunizations not available in the ED setting. Insurance coverage may now have aligned children with a PCP for both wellness and other non-emergent needs. The increase in the proportion of ED visits for newly eligible Medicaid patients (21–65 years of age) observed in this study may reflect the literature regarding barriers to regular primary care in this population and the use of the ED as the safety net. While one major aim of the ACA was to expand the use of primary care, this goal may not have been realized in the study’s population and timeframe.^15^

This study employed the Billings-Ballard algorithm to investigate the severity of ED visits pre- and post-ACA implementation across all age ranges. The severity of ED visits in this study varied by ACA implementation. The decrease in non-emergency visits to this single AMC ED post-implementation would align with the goals of the ACA to decrease potentially unnecessary, non-emergent visit types to the ED. Emergent visits increased post ACA, as did hospitalizations at the AMC. Severity of visits at the CH ED post ACA was not similarly impacted. On the contrary, at the CH, the post-ACA period saw increased odds of non-emergent visits and a statistically significant decline in the ED visits that resulted in hospitalization compared with pre ACA. This disparity in severity of visits across just one healthcare system’s AMC and CH points to the still-incomplete understanding of how our patients use their insurance to access healthcare. Health system allocation of resources across hospitals, patients’ perception regarding need for tertiary care, ED wait times, access to both urgent care and primary care, as well as emergency medical services, may all impact the differences in severity of ED visits between the ACA and the CH over time.

As healthcare systems are called upon to reduce unnecessary costs while still providing value, redirecting non-emergent ED care to less costly alternatives within the system will continue to be prioritized. If emergent visit types also represent high-risk, high-utilization patients, the system should prioritize these patients for care coordination. Incentivizing PCPs to see Medicaid patients in an ambulatory environment has shown to be impactful in improving access for non-emergent conditions in the past and should be investigated again.^16^

## LIMITATIONS

This study analyzed the severity of visits at two Illinois EDs in a major urban area that may not be representative of trends in visits to other health systems’ EDs across the state or country. The geographic span of the study’s institutions include neighborhoods with high poverty levels, and thus our results may reflect the effects of the ACA for low-income individuals. Inclusion of both an urban academic medical center and a community hospital in the study may improve the generalizability of our findings. The unclassified category in our analysis was aligned with that of the Ballard algorithm, in addition to cases involving a primary diagnosis of injury, mental health conditions, alcohol or substance abuse; these may represent a not-insignificant burden of visits to any ED. The CH’s data query was limited to the period when electronic data from the EHR was available at that site, in the beginning of January 2013. The expansion of the local, urgent care networks, as well as the primary care networks related to the AMC and CH, may have impacted ED utilization although neither was analyzed in this study.

## CONCLUSION

In a Medicaid expansion state, the impact of the ACA on a single health system was not consistent across an academic health center and a community hospital. However, a consistent decrease in Medicaid ED visits was observed for children < 18 years and an increase for adults between 21–65 years of age. A larger proportion of ED visits to the AMCs were emergent in the post-ACA period, which was not observed at the CH.

Results from this study may impact the redesign of healthcare reimbursement and delivery systems with an emphasis on preventive and primary care, and an integrated care approach for avoiding preventable ED visits. Our results should not be interpreted for cost-containment measures by health insurers in penalizing patients for presenting to the ED with self-assessed symptoms that could have been serious and by avoiding the ED visit, detrimental to patients’ health.^17^,^18^

## Figures and Tables

**Table 1:** Patient characteristics at a single, suburban health system emergency department before and after the implementation of the Affordable Care Act in Illinois (N=357,764; 2011–2016).

	Academic Medical Center			Community Hospital		
	
Patients characteristics	Pre-Affordable Care Act	Post-Affordable Care Act	p-value	Pre-Affordable Care Act	Post-Affordable Care Act	p-value
Number of visits^a^	143,372	120,881		23,253	70,258	
Gender			<0.001			0.2
Female (%)	53.8	53		55.9	55.4	
Male (%)	46.2	47.0		44.1	44.6	
Age (%)			<0.001			<0.001
< 18 years	24.7	22.3		17.5	16.0	
18 – 20.9 years	3.9	3.5		4.2	4.0	
21 – 25.9 years	7.2	7.1		7.8	8.0	
26 – 39.9 years	17.9	17.5		19.0	19.3	
40 – 64.9 years	28.9	30.9		28.7	29.5	
65 years and older	17.4	18.8		22.9	23.2	
Race (%)			<0.001			0.047
White	40.7	42.7		75.0	74.3	
Black	39.8	36.2		18.5	18.9	
Asian	1.1	1.5		1.2	1.4	
Other^b^	18.4	19.4		5.2	5.2	
Ethnicity^c^(%)			<0.001			0.33
Non-Hispanic	78	74.4		64.2	63.9	
Hispanic	21.7	24.8		35.3	35.7	
Insurance Status(%)			<0.001			<0.001
Private	32.7	31.8		30.1	28.8	
Medicare	22.0	23.4		26.3	26.3	
Medicaid, other governmentally insured	33.2	38.5		29.7	35.1	
Uninsured	12.1	6.4		13.9	9.8	

a Visit numbers pre- and post-Affordable Care Act are not comparable due to different assessment periods.

b Other race categories include Alaska native, Native American, multiracial, Native Hawaiian, and other Pacific Islander.

c Column percents do not total 100% due to missing values in the ethnicity variable. Missing categories were excluded from the bivariate analysis reported in the table.

**Table 2:** The characteristics of emergency department visits before and after the implementation of the Affordable Care Act at a single, suburban health system in Illinois (N = 357,764; 2011–2016).

	Academic Medical Center			Community Hospital		
	
Characteristics of ED visits	Pre-Affordable Care Act	Post-Affordable Care Act	p-value	Pre-Affordable Care Act	Post-Affordable Care Act	p-value
Number of visits/patient/year (mean, SD)	1.41 (1.14)	1.35 (0.98)	<0.001	1.38 (0.95)	1.36 (0.92)	0.16
Readmissions (%)						
Within 48 hours	2.1	2.1	0.75	2.0	1.9	0.3
Within 1 week	5.2	5.0	0.013	4.7	4.7	0.9
Visit resulted in hospitalization (%)	32.1	35.0	<0.001	29.4	28.2	<0.001

**Table 3:** The odds of non-emergent emergency department (ED) visits compared to intermediate and/or emergent ED visits by Affordable Care Act (ACA) implementation status where the pre-ACA period is “referent” category and stratified by insurance status (N = 298,947).

	Odds ratio (OR) of non-emergent vs intermediate and emergent (N= 298,947) OR (95% CI)	Odds ratio of non-emergent vs emergent (N=291,222, excluding intermediate ED visits) OR (95% CI)
Academic Medical Center		
All ED visits	0.68 (0.66–0.70)**	0.66 (0.64–0.67)**
For privately insured	0.68 (0.65–0.71)**	0.66 (0.63–0.69)**
For Medicaid and other government-insured	0.71 (0.68–0.74)**	0.74 (0.71–0.77)**
Medicare	0.66 (0.63–0.70)**	0.65 (0.62–0.69)**
Uninsured or self-pay	0.65 (0.59–0.71)**	0.63 (0.57–0.69)**
Community Hospital		
All ED visits	1.09 (1.04–1.14)**	1.08 (1.03–1.13)**
For privately insured	1.05 (0.97–1.15)	1.04 (0.95–1.13)
For Medicaid and other government-insured	1.03 (0.95–1.13)	1.03 (0.94–1.13)
Medicare	1.11 (1.02–1.21)*	1.10 (1.01–1.20)*
Uninsured or self-pay	1.43 (1.23–1.66)**	1.41 (1.20–1.64)**

*CI*, confidence interval.

Pre-ACA period is the “referent” category. Analyses controlled for insurance status (in the all-ED visits model), age, gender, race, ethnicity and patient-random effects. ED visits were excluded from the analysis if they were unclassifed according to the Ballard algorithm and occurred during the study exclusion period. Missing categories were treated as separate categories.

* p<0.05;

** p<0.001.
